# Operationalizing Primary Outcomes to Achieve Reach, Effectiveness, and Equity in Multilevel Interventions

**DOI:** 10.1007/s11121-023-01613-2

**Published:** 2023-12-04

**Authors:** Kate Guastaferro, R. Christopher Sheldrick, Jillian C. Strayhorn, Emily Feinberg

**Affiliations:** 1 Department of Social and Behavioral Sciences, School of Global Public Health, New York University, New York, NY USA; 2 Department of Psychiatry, University of Massachusetts Chan Medical School, Worcester, MA USA; 3 Hassenfeld Child Health Innovation Institute, Brown University School of Public Health, Providence, RI USA

**Keywords:** Intervention science, Equity, Resource allocation, Multilevel interventions, Simulation

## Abstract

When intervention scientists plan a clinical trial of an intervention, they select an outcome metric that operationalizes their definition of intervention success. The outcome metric that is selected has important implications for which interventions are eventually supported for implementation at scale and, therefore, what health benefits (including how much benefit and for whom) are experienced in a population. Particularly when an intervention is to be implemented in a population that experiences a health disparity, the outcome metric that is selected can also have implications for equity. Some outcome metrics risk exacerbating an existing health disparity, while others may decrease disparities for some but have less effect for the larger population. In this study, we use a computer to simulate implementation of a hypothetical multilevel, multicomponent intervention to highlight the tradeoffs that can occur between outcome metrics that reflect different operationalizations of intervention success. In particular, we highlight tradeoffs between overall mean population benefit and the distribution of health benefits in the population, which has direct implications for equity. We suggest that simulations like the one we present can be useful in the planning of a clinical trial for a multilevel and/or multicomponent intervention, since simulated implementation at scale can illustrate potential consequences of candidate operationalization of intervention success, such that unintended consequences for equity can be avoided.

**Supplementary Information:**

The online version contains supplementary material available at 10.1007/s11121-023-01613-2.

In the design of clinical trials, intervention scientists specify what constitutes a successful result—that is, what outcome the prevention or treatment intervention is intended to produce. When an intervention is expected to produce desirable outcomes on a population level, “intervention success” can be operationalized in a variety of ways. Often, intervention scientists operationalize success in terms of overall mean health outcomes, for example, by setting a goal to reduce mean symptom severity across an entire population. Sometimes, intervention scientists operationalize success in terms of the distribution of health outcomes, for example, by setting a goal to reduce the range of symptom severity observed in the population. How success is operationalized influences every element of the trial from how metrics for primary outcomes are defined to initial power analyses to interpretation of results—including determination of whether an intervention is “effective” and thus worth implementing. By extension, how success is operationalized has important implications for which interventions get delivered at scale, with what benefits, and for whom.

The question of which interventions get delivered at scale—and with what benefits for whom—has important implications for health equity. Equity has long been a priority of many health researchers (Nelson, 2002) attuned to the history of racial injustice and its present consequences (Zambrana & Williams, 2022). Current events and social movements (e.g., Black Lives Matter) have amplified the need for reflection and education (Shim, 2021), changes to policy and funding (Liao et al., 2021; Nundy et al., 2022), and reforms in the delivery of health services (Weine et al., 2020). This has been matched by calls to reform the practice of science (Biglan et al., 2023; Brownson et al., 2021; Kerkhoff et al., 2022; Murry et al., 2022). This paper adds to this growing body of literature by examining implications for health equity when using common metrics (e.g., mean score or Cohen’s *d*) to analyze the success of an intervention in impacting impact health outcomes (e.g., mental health symptoms).

In this study, we use a computer-based simulation of a hypothetical multilevel, multicomponent intervention to demonstrate how choices about the operationalization of intervention success can shape how health benefits are experienced across a population when an intervention is implemented widely (e.g., who experiences change, how much, and to what end post-intervention). A multilevel, multicomponent intervention is one in which two or more levels of influence—for example, child symptoms and clinic-level functioning—are targeted using two or more intervention components—for example, a treatment component and a screening tool. The simulation is designed to inspire further consideration and discussion among investigators designing clinical trials. In particular, we highlight tradeoffs among different types of population benefit under different operationalizations of intervention success, with a particular focus on implications for health equity. We suggest that systematically considering such tradeoffs a priori is a useful exercise for intervention scientists who are designing trials for multilevel and/or multicomponent interventions, especially when those interventions are eventually to be implemented in a population that experiences a health disparity.

## Overview of the Simulation

### Motivating Context

For our computer simulation, we imagine a multilevel treatment and screening intervention to improve the mental health of a population of children. Mental health symptoms are distributed across this hypothetical population in a realistic way: the majority of children have few symptoms, a smaller number of children have more symptoms, and a subset of the population experiences a level of symptom severity that exceeds a diagnostic threshold. Among individuals who exceed this threshold, and are therefore eligible for treatment, symptoms range from mild to severe across 20 different categories of severity. Additionally, we imagine that everyone in this population is a member of one of two readily identifiable subgroups: A and B. On average, subgroup B experiences higher symptom severity than subgroup A; therefore, more of the members of subgroup B are eligible for treatment than subgroup A. Subgroup B also experiences more barriers to accessing care and is, thus, more costly to reach.

The hypothetical intervention includes multiple components. One category of components consists of treatment services that directly improve mental health symptoms on the individual child level. Our simulation assumes that more implementation of treatment services leads to greater “effectiveness”—i.e., a larger health benefit for each child who receives treatment. A second category of intervention components consists of mental health screening services delivered at the clinic level. Our simulation assumes that more implementation of screening services leads to greater “reach”—i.e., a larger proportion of the eligible population receiving treatment. Because all implementation decisions are constrained in practice by resource constraints (Klonschinski, 2014; Weinstein et al., 2009), our simulation constrains implementation of treatment and screening services by imposing a fixed upper limit on implementation cost (in US dollars). Thus, any decision to implement services incurs an opportunity cost (Russell, 1992); that is, the resources that go into implementing certain services cannot be allocated elsewhere (e.g., to deliver other useful services).

### Simulating Implementation

For each iteration of the simulation, the starting point involves operationalizing success for the hypothetical intervention. We imagine that a hypothetical intervention scientist is asked to identify what outcome or combination of outcomes they care about most for the hypothetical population, and we then try all possible combinations of services (treatment services and screening services for subgroup A and for subgroup B) within the fixed cost constraint, identifying the one that maximizes that outcome (or combination of outcomes). We use this to indicate how implementation would proceed, and with what eventual health benefits, depending on the way in which intervention success is defined.

### Operationalizing Intervention Success

We consider three (of many) possible primary outcome metrics to quantify mental health symptom scores. Each represents one answer to the question of how to operationalize intervention success—i.e., what matters most to the hypothetical intervention scientist:

1. Overall population impact, defined as the product of effectiveness and reach (i.e., effectiveness × reach; Glasgow et al., 2001). Because a zero value for either effectiveness or reach means that their product is also zero, this strategy favors a balance of effectiveness and reach. Prioritizing the product of effectiveness and reach on a mean population level also gives priority to those who are relatively easier (i.e., less costly) to reach—in this hypothetical scenario, subgroup A.
2. Proportion of the eligible child population that experiences clinically significant change in mental health symptom severity. Prioritizing this metric similarly defines intervention success in terms of a balance of effectiveness and reach for the overall population—but, in contrast to the first strategy, only enough effectiveness to produce a minimal clinically important difference (MCID). An MCID reflects a patients’ change in score from a clinical intervention that is meaningful for that patient (Cook, 2008). The MCID represents the minimum amount of effectiveness that can be interpreted as clinically meaningful. In this strategy, we constrain effectiveness to be no greater than the MCID, thereby increasing the proportion of the population that can experience at least some degree of benefit. As before, priority is given to those who are relatively easier to reach: subgroup A.
3. Functioning among members of the population who have the highest symptom severity. The priority in this case is the “tail” of the distribution of symptoms in the population after treatment, and the goal is to minimize the burden of mental health problems for children who are most severely affected. Stated differently, this maximizes the minimum outcome. Intervention success, then, is defined as the balance of effectiveness and reach that achieves this end. This strategy prioritizes those in either subgroup with the most severe symptoms but especially the subgroup that experiences greater symptom severity—in this hypothetical scenario, subgroup B.

### Evaluating Performance and Identifying Tradeoffs

Using these three primary outcome metrics—each of which represents a different operationalization of intervention success—we run three separate versions of the simulation, identifying the combination of services that maximizes the identified priority outcomes in each version. Because we evaluate implementation of this hypothetical intervention in simulation, we have the benefit of perfect information about the consequences (i.e., the mental health benefits or any lack thereof) for the simulated population of children. For each simulation version, we report the three outcomes described above: overall population impact, proportion of the eligible child population that experiences clinically significant change, and functioning among those with highest symptom severity. We also report mean symptom severity pre- and post-intervention, and mean symptom severity for the group with the most severe symptoms pre- and post-intervention. We report all of these for the overall population and for the two subgroups, A and B. Comparing results across the three alternative operationalizations of success allows us to identify associated tradeoffs, or types of benefit that could have been achieved but were not, given the specific operationalization of intervention success.

## Methods

### Brief Overview

The context of our simulation is a hypothetical multilevel intervention designed to screen and treat mental health symptoms in children. We simulated two subgroups of a population (A and B)—one of which experiences mental health symptoms that are 20% higher than the other and also costs 20% more to reach (Step 1). Then we simulated implementation of a multilevel intervention that is capable of different levels of effectiveness and reach for these subgroups depending on how success for the intervention is operationalized (Step 2), and we evaluated the resulting health benefits (or lack thereof) for the two subgroups and overall population (Step 3).

### Step 1: Simulating Population Subgroups

Symptoms in subgroup A follow a lognormal distribution, similar to empirical results for the widely used Strengths & Difficulties Questionnaire (mean = 1, standard deviation = 1; Sheldrick et al., 2015). Consistent with estimates from the Centers for Disease Control and Prevention that 1 in 5 children currently or at some point during their life have a seriously debilitating mental illness (Merikangas et al., 2010), we identified the symptom score above which 20% of the simulated children score and used that score to define a hypothetical diagnostic threshold.

Symptoms in subgroup B similarly follow a lognormal distribution but with a mean symptom score that was 20% higher (mean = 1.2; standard deviation = 1). Because subgroup B experiences more mental health symptoms, 26.1% of children in this subgroup scored above the diagnostic threshold. Thus, in the overall simulated population, a greater proportion of the children who meet criteria for intervention are from subgroup B (26.1/(26.1 + 20.0)) than from subgroup A (20.0/(26.1 + 20.0)).

**Fig. 1:**
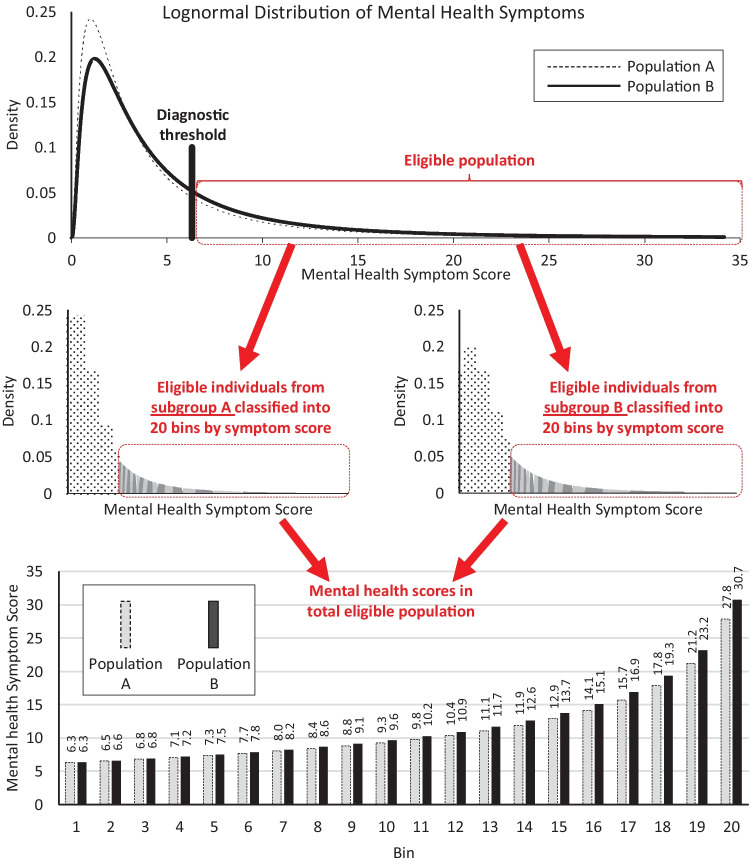
Simulated populations

Within each subgroup, we stratified individual children into 20 “bins” based on their symptoms scores (Fig. 1). Using 20 bins is analogous to a 20-point range on a checklist of mental health symptoms (bins are also referred to as intervals or buckets). Then, in each bin we assigned a mental health score equivalent to the minimum value for the bin to all members of that bin. In subgroup A, the least symptomatic bin had a score of 6.3; the most symptomatic, a score of 27.8. In subgroup B, the least symptomatic bin also had a mean score of 6.3, but the most symptomatic had a mean score of 30.7.

### Step 2: Simulating Implementation of a Hypothetical Multilevel Mental Health Intervention

We define the effectiveness of the hypothetical intervention in terms of a change in symptom severity (lower score indicates fewer symptoms and, thus, is better) and clinically significant change in terms of a MCID of 2 points. Larger effectiveness is accomplished by investing more money into treatment services (e.g., training and quality assurance for cognitive behavioral therapy). We assume that treatment services necessary to affect a 1-point change in symptoms costs $62,500 for patients from subgroup A and $75,000 for patients from subgroup B.

We define reach as the proportion of the eligible population (overall or subgroup-by-subgroup) that receives treatment. Greater reach is accomplished by investing more money into screening services (i.e., such that eligible individuals are identified). We assume that the investment necessary to reach each 1% of the total patient population be $10,000 for patients from subgroup A and $12,000 for patients from subgroup B. In the eligible population that had symptoms that met or exceeded the threshold for eligibility, 43.4% were from subgroup A. Thus, the cost to reach all eligible individuals from subgroup A would be 0.434 × 10,000 = $434,254. In the eligible population, 56.6% were from subgroup B. Thus, the cost to reach all eligible individuals from subgroup B would be 0.566 × 12,000 = $678,895. We let the fixed cost constraint under which the hypothetical investigator must operate equal $1,000,000, and we assume that all dollars must be allocated among four different services: treatment services in subgroup A, screening services in subgroup A, treatment services in subgroup B, and screening services in subgroup B.

In the first-simulation version, the outcome metric used to operationalize intervention success is overall population impact, defined as the product of effectiveness and reach (e.g., Glasgow et al., 1999, 2001) for the eligible population (including children from both subgroups A and B). The most successful intervention is the one that combines treatment and screening services for the subgroups in a manner that maximizes impact in the eligible population. In the second simulation, the outcome metric used to operationalize intervention success is the proportion of the eligible population that experiences a MCID; the most successful intervention is the one that maximizes the proportion of eligible children (from either subgroup A or subgroup B) who experience an improvement in symptom severity of 2.0. In the third simulation, the outcome metric used to operationalize intervention success is mean symptom severity post-intervention among the 5% who experience the most severe symptoms; the most successful intervention is the one that minimizes symptoms for these children, including children from either subgroup A or B.

### Step 3: Identifying Successful Interventions and Evaluating Resulting Health Benefits

To identify the most successful intervention in each simulation version, we ran a grid search of possible allocations to treatment and screening services in subgroups A and B (i.e., all possible interventions composed of some set of services for the two subgroups). A grid search is a method for fine tuning a model in which an exhaustive search of all possible combinations of parameter values is conducted, ultimately arriving at a model producing the most accurate predictions. For example, a grid search might begin by allocating all $1,000,000 to treatment services in subgroup A, then proceed to allocate $997,500 to treatment services in subgroup A and $2,500 to screening services in subgroup A, followed by allocating $995,000 to treatment services in subgroup A and $5,000 to screening services in subgroup B and so on. The grid search proceeds until all allocations have been tested (ending with all $1,000,000 allocated to screening services in subgroup B). Our grid search considered all possible allocations (1,373,701) of the available $1,000,000 (in increments of $2,500) across the four service categories (i.e., treatment services in subgroup A, screening services in subgroup A, treatment services in subgroup B, and screening services in subgroup B). For each allocation, we recorded the priority outcome for that particular simulation version. Any time the grid search tested a new budget allocation that produced a better priority outcome, the computer simulation identified that allocation as the most successful form of the intervention. By considering all possible allocations, we identify the allocation—and thus, the intervention—that yielded the most desirable outcome given the operationalization of intervention success.

When the most successful allocation (intervention combination) was identified under the three operationalizations of intervention success, we evaluated health benefits in the two subgroups and the overall population using the following outcome metrics: (1) mean mental health scale scores pre- and post-intervention; (2) reach; (3) effectiveness; (4) impact (i.e., effectiveness × reach, or the first-priority outcome); (5) the percent that achieved MCID (the second-priority outcome); and (6) the mean mental health scale scores in the most severe 5% (the third-priority outcome).

## Results

**Fig. 2:**
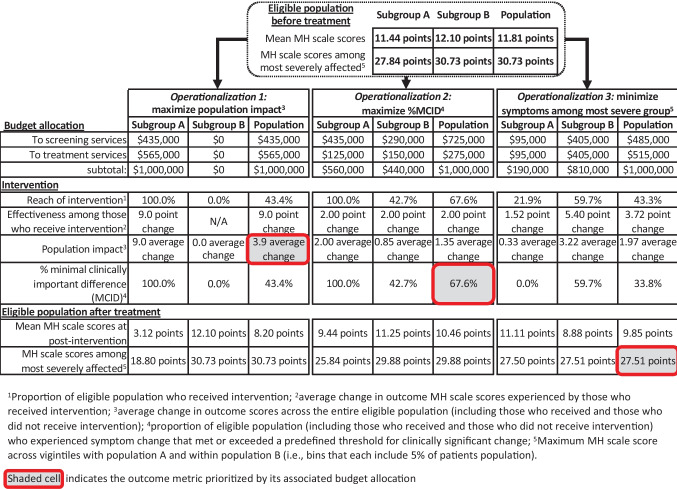
Results of prioritization strategies

Figure 2 summarizes the results for all outcome metrics under each operationalization of intervention success (1 through 3).

### Operationalization 1: Overall Population Impact

Across all combinations of services tested (each reflecting a different budget allocation), the maximum value of population impact was obtained when all resources were allocated to services for subgroup A (see “Budget allocation” for “Operationalization 1” in Fig. 2). Of the $1,000,000 dollars invested into subgroup A, $435,000 went to screening services. This was enough to reach all eligible members of subgroup A (see column for “Subgroup A” under “Operationalization 1” in Fig. 2). The remaining $565,000 went to treatment services, yielding a large 9-point change in mental health scale scores (effectiveness) for those who received treatment in subgroup A. Though no dollars were allocated to support the implementation of the hypothetical intervention in subgroup B, the improvements among subgroup A were enough to improve mean metrics for the overall population. There was a reduction in mean mental health scale scores from 11.8 points at baseline (as indicated in Fig. 1) to 8.2 points (as indicated in the “Population” column under “Operationalization 1” in Fig. 2), resulting in an overall impact (i.e., average change in symptoms across everyone in the eligible population) of 3.9 points (as indicated by the shaded cell under “Operationalization 1” in Fig. 2). That is, when we calculate the average change in symptoms experienced by individuals across the population—including those who experienced no change and those who experienced a 9-point change—the result is 3.9 points. However, the disparity between subgroups A and B increased (see bottom rows of Fig. 2 that report values for “eligible population after treatment”): the mean mental health scale score for subgroup A changed from 11.4 to 3.1, whereas the mean mental health scale score for subgroup B remained constant at 12.1. Moreover, the mean symptom score for the most severe 5% remained at 30.7.

### Operationalization 2: Proportion that Experienced a MCID

Across all combinations of services tested, the maximum proportion of the population to experience MCID was obtained when the intervention contained enough treatment services for each subgroup to bring effectiveness to 2.0 (the MCID). For subgroup A, this represented an investment in treatment services of $125,000 and for subgroup B, $150,000 (see “Budget allocation” for “Operationalization 2” in Fig. 2). The remaining $725,000 were allocated to reach services—$435,000 to reach all eligible children in subgroup A and the remaining $290,000 to reach some of the eligible children in subgroup B. This allocation meant that 67.6% of the overall population experienced a MCID (100% in subgroup A and 42.7% in subgroup B). In the overall population, this allocation was also associated with an improvement in mean mental health scale scores from 11.8 at baseline (as indicated in Fig. 1) to 10.5 (as indicated in the “Population” column under “Operationalization 2” in Fig. 2) and an average impact of 1.35 points for the eligible population. Again, however, the disparity between subgroups and A and B increased. Given preferential allocation to screening services in subgroup A versus B, the difference in means for the two subgroups increased with intervention from 0.7 to 1.8 (i.e., 11.3–9.4), approaching the MCID.

### Operationalization 3: Functioning Among the Most Severe 5%

Across all combinations of services tested, the maximum functioning among the most severe 5% was obtained when the intervention contained more services for subgroup B than subgroup A (representing investments of $810,000 versus $190,000, respectively). Resources were invested into treatment and screening services evenly in both subgroups. In the overall population, implementation of this successful intervention was associated with an improvement in mean mental health scale scores from 11.8 to 9.9 and an average impact of nearly 2.0 points (see “Operationalization 3” in Fig. 2). Subgroup A did not experience much change in mean symptoms; the mean score improved for subgroup A from 11.4 to 11.1. Subgroup B, however, experienced more change, with a mean score that improved from 12.1 to 8.9. The mean symptom scores for the most severe 5% in subgroups A and B also improved—from 27.8 and 30.7, respectively, to 27.5. Thus, the pre-existing disparity between subgroups A and B decreased—or, if quantified in terms of a difference in mean symptoms, was eliminated entirely (leaving subgroup B with better mean functioning than subgroup A).

## Discussion

Increasingly, implementation and intervention scientists are recognizing the importance of equity when evaluating interventions at the population health level (Boyd et al., 2022; Brownson et al., 2021; Shelton et al., 2020). Indeed, a rich theoretical and empirical literature base has posited and documented the notion that health promotion efforts to “control disease” in a population may actually create health disparities (Clouston & Link, 2021; Phelan & Link, 2005; Saldana-Ruiz et al., 2013). This finding amplifies the importance of carefully operationalizing intervention success with equity in mind. In practice, intervention scientists have a variety of operationalizations to choose from when they are designing intervention trials. Eventually, when successful interventions are implemented widely, the implications for equity of choosing a primary outcome metric that operationalizes success in a particular way may become clear in hindsight, but it can be challenging to anticipate these in advance. The use of computer simulation offers one way for intervention scientists to try to anticipate these implications and weigh possible tradeoffs among different types of health benefit, including how much benefit, for whom, and with what effect on the pre-existing health disparity.

In this study, we demonstrate a computer simulation of the implementation of a multilevel, multicomponent mental health intervention comprising some combination of treatment services and screening services for each of two subgroups of children within the population of interest. Implementation influences a variety of outcome metrics, each of which operationalizes a different definition of intervention success. A precise composition of intervention components is selected to maximize one of those metrics, but this can create tradeoffs with other metrics. For example, selecting intervention components that maximize overall population impact can exacerbate disparities in care, whereas selecting intervention components that markedly reduce health disparities may achieve lower values on metrics that reflect overall population impact. Different outcome metrics suggest different ways to determine which operationalization is most desirable (see Supplemental File). For example, Operationalization 1 maximizes population impact; if the intervention scientist prioritizes population impact as an outcome metric, Operationalization 1 would be the best strategy. However, selecting Operationalization 1 involves tradeoffs: although it maximizes population impact, it does not result in as high a proportion of the population achieving MCID as Operationalization 2 and it does not result in functioning among the most severe 5% that is as high as either Operationalization 2 or 3. In short, no one operationalization is superior on all outcome metrics.

By simulating multiple combinations of treatment and screening services, the patterns of health benefits across outcome metrics can be compared. Simulating implementation of an intervention offers one look into what the population-level benefits might be if success for the intervention is defined one way versus another. For this study, we selected three primary outcome metrics that reflect three realistic operationalizations of intervention success: two that were defined in terms of the overall population mean mental health symptoms and one that was defined in terms of the “tail” for observed symptom severity. For each primary outcome metric, the simulation identified the most successful intervention, composed of some set of treatment and screening services for the two subgroups, and estimated various population- and subgroup-level outcomes associated with implementation at scale.

Our results demonstrate how the choice of how to define intervention success—as operationalized by a particular outcome metric—can result in tradeoffs in health benefits. Notably, prioritizing population improvement overall can—at least under some circumstances—increase health disparities in access to services and health outcomes. Thus, selecting an outcome metric that operationalizes a particular definition of intervention success without considering downstream implications risks unintended consequences, including exacerbating inequities. Economists and decision scientists have long recognized that most choices involve tradeoffs, for example, between concepts of efficiency and equity in social policy (Lübbe, 2016; Tinghög, 2016). Our use of simulation modeling to consider potential tradeoffs between different types of population benefit (how much benefit and for whom) may serve as a cautionary tale for those planning a clinical trial. Readers who wish to try out simulation scenarios using our heuristic tool are referred to the Supplementary Files.

Investigators and other interested parties are likely to have different opinions about which outcome metric, and therefore which combination of screening and intervention services, best serves population health. Basic models of decision-making suggest that such differences may derive from (a) different beliefs and expectations about what outcomes are likely to result from different choices and/or (b) values and preferences about which outcomes are most desirable (Hausman, 2011). Our computer simulation eliminates the first source of difference by providing simulated outcomes. However, differences in opinion among decision-makers that result from differences in values and preferences remain possible. Engaging a diverse array of professional and community partners is widely recommended as necessary to achieving health equity (Fleurence et al., 2013; Matthews et al., 2021).

### Limitations

Unlike computer simulations related to study design activities (e.g., power analyses), the simulation described in this paper aimed to facilitate discussions related to the design of trials for multilevel, multicomponent interventions while attending to health equity and, consequently, has different set of limitations. We simulated implementation of a hypothetical intervention in a hypothetical population. While results demonstrate the kinds of tradeoffs in outcomes that are possible, results are not predictive of what will actually occur in a given real-world situation. Moreover, we made a series of simplifying assumptions to demonstrate the use of a simulation model (See Supplemental File A). For example, we assumed that services promoted either reach or effectiveness. In practice, services may increase both effectiveness and reach, as when family navigation increases access to services, thereby improving reach, but family navigators are also trained to deliver problem-solving interventions, which improves effectiveness (Feinberg et al., 2021). Intervention components are likely to have complicated patterns of effectiveness and reach, including variations across individuals and populations that vary by race, ethnicity, culture, and history of marginalization. We also assumed that there was minimal variability in outcomes for different groups within our simulation, something that does not reflect reality but does aid interpretability in the use of the simulation as a simple heuristic.

We considered only three outcome metrics. In practice, a range of other options are commonly reported in the field, such as maximizing the proportion of patients who achieve full symptom remission and relative improvement (which addresses the possibility that the meaning of a 1-point change may differ depending on baseline scores). The field of welfare economics suggest a range of metrics that can be used to measure inequality (Adler, 2022), and to consider how best to aggregate values and preferences at the group level (e.g., a research team; Sen, 2018), rather than just at the individual level (e.g., of a single investigator). In practice, investigators can consider a wide range of metrics that operationalize those definitions.

Finally, while we note that model assumptions should ideally reflect real-world conditions, even for a simple heuristic, relevant data may be uncertain or unavailable—especially with regard to reach and effectiveness in populations that experience disparities in care. Best practice would include sensitivity analyses—i.e., running a model many times under different sets of assumptions to determine under what conditions desired results obtain. Given sufficient evidence and resources to run analyses, model assumptions should ideally reflect real-world conditions—including realistic estimates of variability in outcomes. However, the steps necessary to achieve greater validity of a simulation model come with their own costs, including investigator time and the increased complexity of the resulting model.

## Conclusion

Our simulation illustrates how a single primary outcome (in this case, mental health symptoms) can be quantified using different metrics, and how the choice of metric can have implications for equity. We simulated three different intervention combinations, each of which operationalized a different definition of intervention success, and each of which performed best on a single outcome metric—but less well on others. As such, choice of outcome metric can have implications for health equity. To the extent that different outcome metrics favor different interventions, they influence what is considered to be “evidence-based,” how resources are allocated to different intervention components and to different groups within the larger population, and how the success of interventions is evaluated over time.

How best to resolve the tradeoffs that are identified in a simulation like the one we present—that is, which values should guide our decisions—is a much larger question. Interdisciplinary theory in ethics and social choice offer guidance, but they point to no sure method to resolve these tradeoffs to everyone’s satisfaction. If the arc of history is truly to bend toward justice (Dreyer et al., 2020), deeper engagement with one another—both professional and community partners alike—may offer the best path to prioritizing outcome metrics for the interventions we design, test, implement, and scale with the goal of improving population health.

### Supplementary Information

Below is the link to the electronic supplementary material.

Supplementary file1 (DOCX 1271 KB)

Supplementary file2 (DOCX 356 KB)

## References

[CR1] Adler, M. D. (2022). Theory of prioritarianism. In M. D. Adler & O. F. Norheim (Eds.), *Prioritarianism in Practice* (pp. 37–127). Cambridge University Press. 10.1017/9781108691734.002

[CR2] Biglan, A., Prinz, R. J., & Fishbein, D. (2023). Prevention science and health equity: A comprehensive framework for preventing health inequities and disparities associated with race, ethnicity, and social class. *Prevention Science: The Official Journal of the Society for Prevention Research*, 1–11. 10.1007/s11121-022-01482-1 10.1007/s11121-022-01482-1 PMC10227139 36757658

[CR3] Boyd, R. C., Castro, F. G., Finigan-Carr, N., Okamoto, S. K., Barlow, A., Kim, B.-K. E., Lambert, S., Lloyd, J., Zhang, X., Barksdale, C. L., Crowley, D. M., Maldonado-Molina, M., Obasi, E. M., & Kenney, A. (2022). Strategic directions in preventive intervention research to advance health equity. *Prevention Science: The Official Journal of the Society for Prevention Research*, 1–20. 10.1007/s11121-022-01462-5 10.1007/s11121-022-01462-5 PMC9734404 36469162

[CR4] Brownson RC, Kumanyika SK, Kreuter MW, Haire-Joshu D (2021). Implementation science should give higher priority to health equity. Implementation Science.

[CR5] Clouston SAP, Link BG (2021). A retrospective on fundamental cause theory: State of the literature and goals for the future. Annual Review of Sociology.

[CR6] Cook CE (2008). Clinimetrics corner: The Minimal Clinically Important Change Score (MCID): A necessary pretense. The Journal of Manual & Manipulative Therapy.

[CR7] Dreyer, B. P., Trent, M., Anderson, A. T., Askew, G. L., Boyd, R., Coker, T. R., Coyne-Beasley, T., Fuentes-Afflick, E., Johnson, T., Mendoza, F., Montoya-Williams, D., Oyeku, S. O., Poitevien, P., Spinks-Franklin, A. A. I., Thomas, O. W., Walker-Harding, L., Willis, E., Wright, J. L., Berman, S., & Stein, F. (2020). The death of George Floyd: Bending the arc of history toward justice for generations of children. *Pediatrics*, *146*(3), e2020009639. 10.1542/peds.2020-009639 10.1542/peds.2020-009639 32591435

[CR8] Feinberg E, Augustyn M, Broder-Fingert S, Bennett A, Weitzman C, Kuhn J, Hickey E, Chu A, Levinson J, Sandler Eilenberg J, Silverstein M, Cabral HJ, Patts G, Diaz-Linhart Y, Rosenberg J, Miller JS, Guevara JP, Fenick AM, Blum NJ (2021). Effect of family navigation on diagnostic ascertainment among children at risk for autism: A randomized clinical trial from DBPNet. JAMA Pediatrics.

[CR9] Fleurence R, Selby JV, Odom-Walker K, Hunt G, Meltzer D, Slutsky JR, Yancy C (2013). How the Patient-Centered Outcomes Research Institute is engaging patients and others in shaping its research agenda. Health Affairs (project Hope).

[CR10] Glasgow RE, McKay HG, Piette JD, Reynolds KD (2001). The RE-AIM framework for evaluating interventions: What can it tell us about approaches to chronic illness management?. Patient Education and Counseling.

[CR11] Glasgow RE, Vogt TM, Boles SM (1999). Evaluting the public health impact of health promotion interventions: The RE-AIM framework. American Journal of Public Health.

[CR12] Hausman, D. M. (2011). *Preference, value, choice, & welfare*.

[CR13] Kerkhoff AD, Farrand E, Marquez C, Cattamanchi A, Handley MA (2022). Addressing health disparities through implementation science—A need to integrate an equity lens from the outset. Implementation Science.

[CR14] Klonschinski A (2014). “Economic imperialism” in health care resource allocation—How can equity considerations be incorporated into economic evaluation?. Journal of Economic Methodology.

[CR15] Liao JM, Lavizzo-Mourey RJ, Navathe AS (2021). A national goal to advance health equity through value-based payment. JAMA.

[CR16] Lübbe W, Nagel E, Laurer M (2016). Social value maximation and the multiple goals assumption: Is priority setting a maximizing task at all?. Prioritization in Medicine.

[CR17] Matthews K, Morgan I, Davis K, Estriplet T, Perez S, Crear-Perry JA (2021). Pathways to equitable and antiracist maternal mental health care: Insights from black women stakeholders. Health Affairs (project Hope).

[CR18] Merikangas, K. R., He, J.-P., Burstein, M., Swanson, S. A., Avenevoli, S., Cui, L., Benjet, C., Georgiades, K., & Swendsen, J. (2010). Lifetime prevalence of mental disorders in U.S. adolescents: Results from the National Comorbidity Survey Replication—Adolescent Supplement (NCS-A). *Journal of the American Academy of Child & Adolescent Psychiatry*, *49*(10), 980–989. 10.1016/j.jaac.2010.05.017 10.1016/j.jaac.2010.05.017 PMC2946114 20855043

[CR19] Murry VM, Bradley C, Cruden G, Brown CH, Howe GW, Sepùlveda M-J, Beardslee W, Hannah N, Warne D (2022). Re-envisioning, retooling, and rebuilding prevention science methods to address structural and systemic racism and promote health equity. Prevention Science.

[CR20] Nelson A (2002). Unequal treatment: Confronting racial and ethnic disparities in health care. Journal of the National Medical Association.

[CR21] Nundy S, Cooper LA, Mate KS (2022). The quintuple aim for health care improvement: A new imperative to advance health equity. JAMA.

[CR22] Phelan, J. C., & Link, B. G. (2005). Controlling disease and creating disparities: A fundamental cause perspective. *The Journals of Gerontology: Series B*, *60*(Special_Issue_2), S27–S33. 10.1093/geronb/60.Special_Issue_2.S27 10.1093/geronb/60.special_issue_2.s27 16251587

[CR23] Russell LB (1992). Opportunity costs in modern medicine. Health Affairs.

[CR24] Saldana-Ruiz N, Clouston SAP, Rubin MS, Colen CG, Link BG (2013). Fundamental causes of colorectal cancer mortality in the United States: Understanding the importance of socioeconomic status in creating inequality in mortality. American Journal of Public Health.

[CR25] Sen A (2018). Collective choice and social welfare.

[CR26] Sheldrick RC, Benneyan JC, Kiss IG, Briggs-Gowan MJ, Copeland W, Carter AS (2015). Thresholds and accuracy in screening tools for early detection of psychopathology. Journal of Child Psychology and Psychiatry.

[CR27] Shelton, R. C., Chambers, D. A., & Glasgow, R. E. (2020). An extension of RE-AIM to enhance sustainability: Addressing dynamic context and promoting health equity over time. *Frontiers in Public Health*, *8*. 10.3389/fpubh.2020.00134 10.3389/fpubh.2020.00134 PMC7235159 32478025

[CR28] Shim RS (2021). Dismantling structural racism in psychiatry: A path to mental health equity. American Journal of Psychiatry.

[CR29] Tinghög, G. (2016). Health-care priority setting in practice: Seven unresolved problems. In E. Nagel & M. Lauerer (Eds.), *Prioritization in Medicine* (pp. 101–109). Springer, Cham. 10.1007/978-3-319-21112-1_8

[CR30] Weine S, Kohrt BA, Collins PY, Cooper J, Lewis-Fernandez R, Okpaku S, Wainberg ML (2020). Justice for George Floyd and a reckoning for global mental health. Global Mental Health.

[CR31] Weinstein MC, Torrance G, McGuire A (2009). QALYs: The basics. Value in Health.

[CR32] Zambrana RE, Williams DR (2022). The intellectual roots of current knowledge on racism and health: Relevance to policy and the national equity discourse. Health Affairs.

