# Supplementary material for: Operationalizing Primary Outcomes to Achieve Reach, Effectiveness, and Equity in Multilevel Interventions
Source: Prev Sci. 2023 Dec 4;25(Suppl 3):397–406. doi: 10.1007/s11121-023-01613-2 (PMC11239781; doi:10.1007/s11121-023-01613-2)
Supplement: Supplementary file 1 — Supplementary file1 (DOCX 1271 KB) [file 11121_2023_1613_MOESM1_ESM.docx]

**Supplement: Sensitivity Analyses**

To explore the influence of model assumptions on results, we ran 11 additional analyses in which we systematically varied:

- the proportion of the total population that derives from the subpopulation with higher symptoms (25%, 50%, and 75%)
- the cost of services in the subpopulation with higher symptoms compared to the subpopulation with lower symptoms (equivalent, 10% higher, 20% higher, and 30% higher).

A detailed Figure equivalent to Figure 2 in the primary manuscript was prepared for each simulation. Each figure reports a larger number of outcomes, thus precluding any simple summary of them all. However, we do provide summaries of:

- the proportion of the total budget allocated to the subpopulation with higher symptoms;
- the average population impact on the subpopulation with lower symptoms, the subpopulation with higher symptoms, and on the total population.

In addition, we provide:

- a summary of results with respect to population impact on p 2 (Table S1)
- a summary of results with respect to minimal clinically important difference (MCID) on p 3 (Table S2)
- a summary of results with respect to minimizing symptoms among the most severely affected on p 4 (Table S3)

Figures for each individual scenario are as follows:

|  |  | % of total population that derives from subpopulation with higher symptoms | | |
| --- | --- | --- | --- | --- |
|  |  | 25% | 50% | 75% |
| compared to the subpopulation with lower symptoms (A), costs in the subpopulation with higher symptoms (B) are: | the same | Figure S4  (page 5) | Figure S8  (page 9) | Figure S11  (page 12) |
|  | 10% higher | Figure S5  (page 6) | Figure S9  (page 10) | Figure S12  (page 13) |
|  | 20% higher | Figure S6  (page 7) | base case  (see main article, Figure 2) | Figure S13  (page 14) |
|  | 30% higher | Figure S7  (page 8) | Figure S10  (page 11) | Figure S14  (page 15) |

Within each scenario (e.g., set of assumptions regarding relative cost and proportion of total population), results are provided for the budget allocation that maximizes population impact in Table S1. Results demonstrate that if all other assumptions are held constant, population impact is maximized either by allocating 100% of funds to the population with lower symptoms or by allocating 100% of funds to the population with higher symptoms—i.e., intermediate options that portion the available budget between both populations did not maximize overall population impact in the scenarios considered. Moreover, allocating the available budget to the population with higher symptoms was more likely to maximize population impact if that population was in the majority and/or if costs were not higher.

| Table S1. Budget allocated to maximize population impact | | | | |
| --- | --- | --- | --- | --- |
| *Proportion of budget allocated to subpopulation with higher symptoms (A)* | | | | |
|  |  | % of total population that derives from  subpopulation with higher symptoms | | |
|  |  | 25% | 50% | 75% |
| compared to the subpopulation with lower symptoms (A), costs in the subpopulation with higher symptoms (B) are: | the same | 0% | 100% | 100% |
|  | 10% higher | 0% | 0% | 100% |
|  | 20% higher | 0% | 0% | 100% |
|  | 30% higher | 0% | 0% | 0% |
|  |  |  |  |  |
|  |  |  | | |
| *Impact in subpopulation with lower symptoms (A), subpopulation with higher symptoms (B), and total population* | | | | |
|  |  | % of total population that derives from  subpopulation with higher symptoms | | |
|  |  | 25% | 50% | 75% |
| compared to the subpopulation with lower symptoms (A), costs in the subpopulation with higher symptoms (B) are: | the same | 5.7, 0, 4 | 0, 7.1, 4 | 0, 5, 4 |
|  | 10% higher | 5.7, 0, 4 | 9, 0, 3.9 | 0, 4.2, 3.3 |
|  | 20% higher | 5.7, 0, 4 | 9.0, 0, 3.9 | 0, 3.5, 2.8 |
|  | 30% higher | 5.7, 0,4 | 9, 0, 3.9 | 12.7, 0, 2.6 |

Results are provided for the allocation that maximizes the proportion of the population that experiences minimal clinically significant difference (MCID) in Table S2. Results demonstrate that if all other assumptions are held constant, MCID is maximized by allocating anywhere between 18% and 100% of available budget to the population with higher symptoms. Allocating the available budget to the population with higher symptoms was more likely to maximize MCID if that population was in the majority and/or if costs were not higher. When the population that experienced higher symptoms were in the minority (25%) and costs were higher (by 10-20%) than in the population with lower symptoms, it received lower budget allocations (18%) when MCID was maximized.

| Table S2. Budget allocated to maximize the proportion of the population that experiences MCID | | | | |
| --- | --- | --- | --- | --- |
| *Proportion of budget allocated to subpopulation with higher symptoms (A)* | | | | |
|  |  | % of total population that derives from  subpopulation with higher symptoms | | |
|  |  | 25% | 50% | 75% |
| compared to the subpopulation with lower symptoms (A), costs in the subpopulation with higher symptoms (B) are: | the same | 43% | 70% | 100% |
|  | 10% higher | 18% | 44% | 100% |
|  | 20% higher | 18% | 44% | 100% |
|  | 30% higher | 18% | 44% | 100% |
|  |  |  |  |  |
|  |  |  | | |
| *Impact in subpopulation with lower symptoms (A), subpopulation with higher symptoms (B), and total population* | | | | |
|  |  | % of total population that derives from  subpopulation with higher symptoms | | |
|  |  | 25% | 50% | 75% |
| compared to the subpopulation with lower symptoms (A), costs in the subpopulation with higher symptoms (B) are: | the same | 1.3 ,2.1 ,1.5 | 0.8 ,2.1 ,1.5 | 0 ,3.2 ,2.5 |
|  | 10% higher | 2.1 ,0.2 ,1.5 | 2.1 ,1 ,1.4 | 0 ,2 ,1.6 |
|  | 20% higher | 2.1 ,0.1 ,1.5 | 2.0, 0.9, 1.4 | 0 ,1.8 ,1.5 |
|  | 30% higher | 2.1 ,0.1 ,1.5 | 2.1 ,0.7 ,1.3 | 0 ,1.6 ,1.3 |

Results are provided for the allocation that minimizes symptoms among the most severely affected in Table S3. Results demonstrate that if all other assumptions are held constant, this outcome is achieved by allocating anywhere between 56% and 99% of available budget to the population with higher symptoms. Allocating the available budget to the population with higher symptoms was more likely to achieve this outcome if that population was in the majority and/or if costs were higher. When the population that experienced higher symptoms were in the minority (25%) and costs were the same as in the population with lower symptoms, it received the lowest budget allocations (56%) of all scenarios considered.

| Table S3. Budget allocated to minimize symptoms among most severely affected | | | | |
| --- | --- | --- | --- | --- |
| *Proportion of budget allocated to subpopulation with higher symptoms (A)* | | | | |
|  |  | % of total population that derives from  subpopulation with higher symptoms | | |
|  |  | 25% | 50% | 75% |
| compared to the subpopulation with lower symptoms (A), costs in the subpopulation with higher symptoms (B) are: | the same | 56% | 72% | 83% |
|  | 10% higher | 60% | 77% | 88% |
|  | 20% higher | 64% | 81% | 93% |
|  | 30% higher | 67% | 86% | 99% |
|  |  |  |  |  |
|  |  |  | | |
| *Impact in subpopulation with lower symptoms (A), subpopulation with higher symptoms (B), and total population* | | | | |
|  |  | % of total population that derives from  subpopulation with higher symptoms | | |
|  |  | 25% | 50% | 75% |
| compared to the subpopulation with lower symptoms (A), costs in the subpopulation with higher symptoms (B) are: | the same | 1.1 ,4 ,2 | 0.7 ,3.6 ,2.4 | 0.6 ,3.5 ,2.9 |
|  | 10% higher | 1.1 ,3.3 ,1.8 | 0.5 ,3.4 ,2.1 | 0.3 ,3.2 ,2.6 |
|  | 20% higher | 0.8 ,3.7 ,1.6 | 0.3, 3.2, 2.0 | 3 ,2.4 ,2.4 |
|  | 30% higher | 0.6 ,3.5 ,1.5 | 0.2 ,3.1 ,1.8 | 0 ,2.9 ,2.3 |
