# Supplementary material for: Operationalizing Primary Outcomes to Achieve Reach, Effectiveness, and Equity in Multilevel Interventions
Source: Prev Sci. 2023 Dec 4;25(Suppl 3):397–406. doi: 10.1007/s11121-023-01613-2 (PMC11239781; doi:10.1007/s11121-023-01613-2)
Supplement: Supplementary file 2 — Supplementary file2 (DOCX 356 KB) [file 11121_2023_1613_MOESM2_ESM.docx]

**Simulation Assumption Documentation**

I. Policy scenario. The simulator assumes that the decision-maker is concerned with deploying two types of services:

1. Services that promote intervention reach (e.g., population screening, patient navigation)
2. Services that promote intervention effectiveness (e.g., clinician training, quality assurance)

The decision-maker is concerned with deploying these services across two populations. In population A (see Figure S1), mental health symptoms follow a log-normal distribution with a given mean and standard deviation (SD). A clinical threshold is defined as the symptom level at or above which a proportion of the population equivalent to the prevalence scores. Individuals with a score at or above this threshold are considered to have a mental health disorder. Individuals with mental health disorders are stratified into bins. All individuals in a given bin are assigned the same mental health symptom score, which is equivalent to the minimum score for that bin.

The cost to reach each 1% of the population with an intervention and a cost for effectiveness to achieve a 1-point change among patients who receive intervention.

In population B, mental health symptoms also follow a log-normal distribution, but the mean and SD can vary. In our baseline scenario, mean mental health scores are 20% higher in population B compared to population A (see Figure S2). Costs in population B are equivalent to costs in population A multiplied by a cost ratio (B:A).

The model tracks whether intervention causes a change in mental health symptoms greater than or equal to a pre-defined clinically significant difference. It also tracks whether absolute symptoms scores after treatment fall below a threshold for remission.

Inputs for this policy scenario can be defined by the user. Table 1 reports base case values for each input.

| Table 1. Inputs for policy scenario | | |
| --- | --- | --- |
| **Input** | **Value in baseline scenario** | **Notes** |
| Mean_A | 1 | Mean of log-normal distribution of mental health symptoms in population A |
| SD_A | 1 | SD of log-normal distribution of mental health symptoms in population A |
| Prevalence_A | 20% | Prevalence in population A |
| Bins | 20 | Number of levels into which individuals in each population who have mental health disorders are stratified |
| Cost for reach (cost_reach_A) | $10,000 | Cost to reach each 1% of the population with an intervention |
| Cost for effectiveness (cost_effectiveness_A) | $62,500 | Cost to achieve a 1-point change among patients who receive intervention |
| Mean_B | 1 | Mean of log-normal distribution of mental health symptoms in population B |
| SD_B | 1 | SD of log-normal distribution of mental health symptoms in population B |
| Cost ratio (B:A) | 1.2 | Costs in population B compared to population A |
| Threshold for remission | 5.5 | Threshold below which final symptoms scores must fall to be defined as in “full remission” |
| Clinically significant difference (clinSigDiff) | 2 | Change in mental health scores necessary to be defined as a “clinically significant change” |
| % of total population | 50% | % of total population comprised by population A |

II. Simulated policy decision. Based on the policy scenario outlined above, a policy decision is simulated as follows. The decision-maker has a budget of $1,000,000 that must be allocated among four different sets of services:

1. Services that promote reach in population A (spending_reach_A)
2. Services that promote effectiveness in population A (spending_effectiveness_A)
3. Services that promote reach in population B (spending_reach_B)
4. Services that promote effectiveness in population B (spending_effectiveness_B)

For each completed allocation decision, outcomes are calculated for population A, for population B, and for the overall population.

III. Calculation of outcomes based on simulated policy decision. Based on user inputs (indicated by variable names that are underlined), proximal and outcomes variables (indicated by variable names that are **bold and underlined**) in two populations are estimated as follows:

- *Diagnostic threshold*. Estimates a **diagnostic threshold** equivalent to the score of a lognormal distribution (with mean = mean_A and standard deviation = SD_A) above which a proportion of the population equivalent to prevalence scores
- *Prevalence in population B*. This **diagnostic threshold** is applied to population B, which is defined by a lognormal distribution (with mean = mean_B and standard deviation = SD_B). Prevalence in population B (**prevalence_B**) is estimated as the proportion of population B that meets or exceeds this threshold.
- *Eligible populations A and B*. The total eligible population is comprised of prevalence_A and **prevalence_B**. The proportion of the total patient population from population A (**percentA**) is defined as prevalence_A/( prevalence_A+ **prevalence_B).** The proportion of the total patient population from population B (**percentB**) is defined as **prevalence_B**/( prevalence_A+ **prevalence_B).**
- *Costs in population B*. Cost for reach in population B (**cost_reach_B**) is defined as cost_reach_A*Cost_ratio(A:B). Cost for effectiveness in population B (**cost_effectiveness_B**) is defined as cost_effectiveness_A*Cost_ratio(A:B).
- *Reach*. Reach in population A (**Reach_A**) is defined as spending_reach_A/(**percentA***100*cost_reach_A). Reach in population B (**Reach_B**) is defined as spending_reach_B/(**percentB***100*cost_reach_B).
- *Effectiveness*. Effectiveness in population A (**Effectiveness_A**) is defined as spending_effectiveness_A/cost_effectiveness_A. Effectiveness in population B (**Effectiveness_B**) is defined as **spending_effectiveness_B**/**cost_effectiveness_B**.
- *Impact*. In population A, impact (**impact_A**) is defined as **reach_A*****effectiveness_A**. In population B, impact (**impact_B**) is defined as **reach_B*****effectiveness_B**. In the overall population, **impact** is defined as **impact_A*percentA + impact_B*percentB**
- *Baseline scores across bins*. Within each population (A & B), eligible individuals (whose MH scale scores exceed **diagnostic threshold**) are divided into a number of groups as defined by Bins. All members of each group are assigned a symptom scale score equivalent to the minimum value for that group. Within population A, this score is referred to as **preScore_A**. Within population B, this score is referred to as **preScore_B**.
- *Scores among individuals who receive intervention*. In each bin of population A, scores at post for those who receive intervention (**postscore_Atx**) are defined as the maximum of zero and **preScore_A- effectiveness_A** (i.e., scores at post cannot be below zero). In each bin of population B, scores at post for those who receive intervention (**postscore_Btx**) are defined as the maximum of zero and **preScore_B- effectiveness_B** (i.e., scores at post cannot be below zero).
- *Mean score in each bin at post*. Intervention reach is distributed equally across all bins. Thus, the proportion of individuals in each bin who receive treatment is defined by **reach_A** in population A and **reach_B** in population B. In each bin of population A, mean score at post (**postscore_A**) is defined as **reach_A*** **postscore_Atx** + (1-**reach_A**)* **preScore_A**. In each bin of population B, mean score at post (**postscore_B**) is defined as **reach_B*** **postscore_Btx** + (1-**reach_B**)* **preScore_B**.
- *Scores among most severely affected*. In population A, the score among the most severely affected (**scoreAmongMostSeverelyAffected_A**) is the maximum value of **postscore_A** across bins. In population B, the score among the most severely affected (**scoreAmongMostSeverelyAffected_B**) is the maximum value of **postscore_B** across bins. In the overall population, the score among the most severely affected (**scoreAmongMostSeverelyAffected**) is the maximum value of **postscore_A** and **postscore_B** across bins.
- *Mean population score at baseline*. In population A, mean score before intervention (**meanPre_A**) is defined as the mean of **preScore_A** across all bins. In population B, mean score before intervention (**meanPre_B**) is defined as the mean of **preScore_B** across all bins. In the overall population, the mean score before intervention is defined as **meanPre_A*****percentA** + **meanPre_B*****percentB**
- *Mean population score at post*. In population A, mean score after intervention (**meanPost_A**) is defined as the mean of **postScore_A** across all bins. In population B, mean score before intervention (**meanPost_B**) is defined as the mean of **postScore_B** across all bins. In the overall population, the mean score before intervention is defined as **meanPost_A*****percentA** + **meanPost_B*****percentB**
- *Clinically significant change*. If **effectiveness_A** >= clinSigDiff, then the proportion of individual in population A who experience a clinically significant change **(%clinSigDiff_A**) = **reach_A**. If **effectiveness_B** >= clinSigDiff, then the proportion of individual in population A who experience a clinically significant change **(%clinSigDiff_B**) = **reach_B**.
